# Neuroinflammation and mental health outcomes in adolescents living with HIV

**DOI:** 10.1097/COH.0000000000000877

**Published:** 2024-07-10

**Authors:** Arish Mudra Rakshasa-Loots, Jaime H. Vera, Barbara Laughton

**Affiliations:** a Edinburgh Neuroscience, School of Biomedical Sciences, The University of Edinburgh, Edinburgh; b Department of Global Health and Infection, Brighton & Sussex Medical School, University of Sussex, Brighton, UK; c Family Centre for Research with Ubuntu (FAMCRU), Department of Paediatrics and Child Health, Stellenbosch University, Cape Town, South Africa

**Keywords:** adolescents with perinatally-acquired HIV, bipolar disorder, major depressive disorder, microglial activation, neuroimaging, panic disorder, schizophrenia, systemic inflammation

## Abstract

**Purpose of review:**

Adolescents living with HIV show chronic inflammation, which in turn has been linked to mental health outcomes in the general population. The increased risk for mental health issues in adolescents with HIV may thus be driven by HIV-related inflammation. In this review, we discuss the associations between peripheral and central nervous system inflammation and mental health outcomes in adolescents with HIV.

**Recent findings:**

Preclinical models indicate that expression of HIV viral proteins early in life may lead to neuroinflammation and behavioural deficits in adolescence. Clinical evidence is available primarily in the general population and in adults with HIV, and suggests that inflammatory biomarkers such as IL-6 and TNF-α may be associated with depressive symptoms. Only one study has explored these relationships in adolescents with HIV, and did not find that inflammatory biomarkers in the blood or brain were linked to depressive symptoms. Current research in this field focuses overwhelmingly on peripheral inflammatory biomarkers (compared to neuroimaging biomarkers) and on depression (compared to other mental health conditions).

**Summary:**

There is strong evidence to suggest that neuroinflammation and peripheral inflammation may play a role in the development of mental health issues in adolescents, but research in adolescents with HIV is sparse. Characterizing the relationship between inflammation and mental health in adolescents with HIV may help improve the prediction, prevention, early intervention, and treatment of mental health issues in this population.

## INTRODUCTION

A substantial proportion of children and adolescents living with HIV experience mental health issues [1,2]. These young people with HIV also exhibit chronic inflammation, evidenced by increased cytokine release, monocyte activation, and alterations in neurometabolites such as choline and myo-inositol, which persists despite successful antiretroviral therapy (ART) [3,4]. Children and adolescents who are exposed to HIV (and ART) *in utero,* but who are not with HIV (often referred to in the literature as ‘HEU’ – HIV Exposed but Uninfected), also show evidence of persistent neuroinflammation [5,6] and worse neurodevelopmental outcomes at two years of age [7]. In the general population, there is some evidence that young people who show inflammation early in life are at greater risk for developing mental health issues. [8]. For instance, an investigation in national registries in Finland identified a significantly higher incidence of psychiatric morbidity among children with juvenile idiopathic arthritis (JIA) compared to controls [9]. Thus, it is possible that the greater risk for mental health issues among young people with HIV may also be driven by peripheral and central nervous system (CNS) inflammation.

Adolescents with HIV face a complex set of psychosocial and biological risk factors for mental health issues, which makes it both challenging and important to characterize the contribution of HIV-related inflammation to these outcomes. Adolescents with HIV are likely to experience poverty, migration or displacement, adverse childhood experiences, and loss of parents or other family members, resulting in increased risk for mental health issues [10]. HEU adolescents experience maternal immune activation and exposure to ART or inflammation *in utero*, which may also possibly contribute to mental health outcomes [11,12^▪^,13^▪^]. Perinatally-acquired HIV is also associated with increased rates of preterm birth, low birth weight, HIV encephalopathy in infancy, and opportunistic infections in the CNS or the periphery, resulting in neuroinflammation and thus potentially making it difficult to discern the precise role of HIV-related inflammation in mental health outcomes [14,15]. Finally, in adolescents with recently-acquired (as opposed to perinatally-acquired) HIV, neuroinflammation during critical neurodevelopmental periods, together with psychosocial factors such as stigma or social isolation, may lead to increased risk for neuropsychiatric conditions [16]. Therefore, adolescents living with (or exposed to) HIV are uniquely vulnerable to neuroinflammation and mental health issues, and whether these risks are linked to each other remains to be established.

In this article, we review recent preclinical and clinical evidence for a relationship between inflammation and mental health issues (with a focus on depression and anxiety disorders) in adolescents, highlighting studies involving adolescents with HIV wherever such data is available. 

**Box 1:**
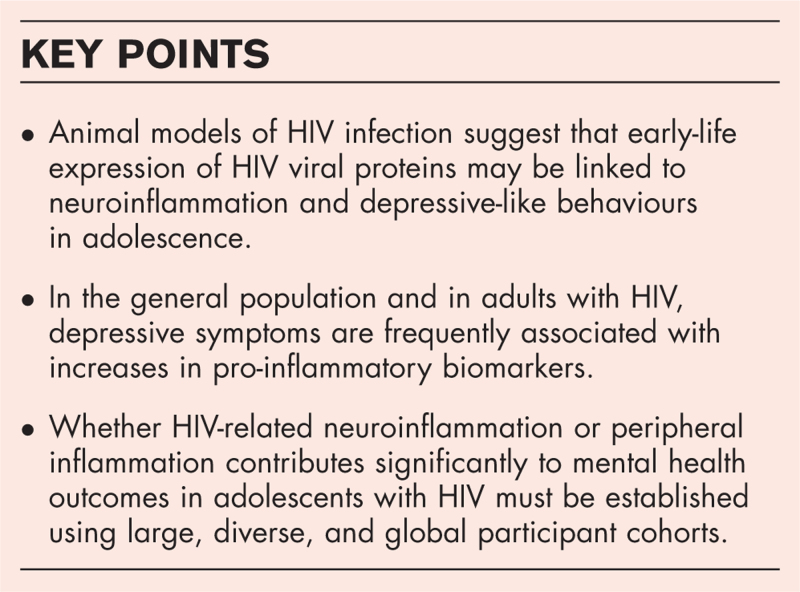
no caption available

## PRECLINICAL EVIDENCE

Preclinical models suggest that HIV viral protein expression during development may result in depression-like phenotypes in adolescence. Animal models of HIV infection can allow for direct measurement of neuroinflammation in brain tissue, which is not often possible in humans. HIV-1 transgenic (Tg) rodents are employed to model ART-controlled HIV infection, since these animals express HIV viral proteins without viral replication. Rowson *et al.*[17] have shown that adolescent HIV-1 Tg rats exhibit behavioural deficits, including decreased central tendency in the open field test (widely considered a measure of anxiety-like behaviour in rodents) and decreased investigative behaviour in the novel object recognition task. These behavioural alterations may thus be linked to the expression of HIV-1 viral proteins during development. Nemeth *et al.*[18] also found that HIV-1 viral protein expression during development resulted in depressive-like behaviours in adolescents HIV-1 Tg rats, along with an increase in gene expression of the chemokine *Mcp-1* in the hippocampus, indicative of neuroinflammation. However, blocking this expression did not rescue depressive-like behaviours in this model, suggesting that *Mcp-1* expression in the hippocampus is not a mediating mechanism for depressive-like behaviours in this HIV-1 Tg model.

In general (non-HIV) animal models, Cao *et al.*[19] have demonstrated that male mice injected with lipopolysaccharide (LPS, an inflammatory compound) at postnatal day 14 exhibited depressive-like behaviours in adolescence. Inhibition of microglia in LPS-injected mice prevented the depressive phenotype, suggesting that microglial activity may mediate the relationship between early life inflammation and depression in adolescence. Cao *et al.*[19] also found that acute stress experienced by LPS-injected mice in adolescence led to increased microglial activation. However, Rowson *et al.*[17] demonstrated that HIV-1 Tg rats exhibited microglial activation, and the experience of chronic stress in adolescence resulted in increased microglial activation in wild-type rats, but no further increases in HIV-1 Tg rats. Therefore, some preclinical evidence suggests that neuroinflammation may mediate the impact of early-life inflammation on depressive-like behaviours in adolescent rodents, and HIV viral protein expression during development may influence this phenotype more than stress experienced later in life.

## CLINICAL EVIDENCE: INFLAMMATION AND ADOLESCENT MENTAL HEALTH

A growing body of clinical evidence in the general population suggests that adolescents with mental health issues may exhibit inflammation. An early systematic review showed increased pro-inflammatory biomarkers in children and adolescents with a range of mental health conditions, including depression, bipolar disorder, and schizophrenia, compared to those without mental health conditions [20]. Dysregulation in inflammatory cytokines such as interleukin (IL)-6 and tumour necrosis factor (TNF)-α has been observed in adolescents with first-episode schizophrenia, bipolar disorder, and depression [21]. This study also observed higher CRP concentrations in young people with bipolar disorder (compared to controls and those with other psychiatric conditions), but another study found no significant associations of CRP concentrations with severity of neuropsychiatric symptoms across a range of conditions [22]. A larger study involving data from the Avon Longitudinal Study of Parents and Children (ALSPAC) also found higher IL-6 and suPAR (considered a biomarker of chronic immune activation) in blood plasma for young adults (aged 24) with psychotic disorder [23]. A recent study involving young people with perinatally-acquired HIV found that emotional symptoms (broadly defined) were associated with brain structural network integrity measures, which in turn were associated with peripheral inflammatory biomarkers [24^▪^]. Together, these findings indicate that mental health issues in young people may be linked to peripheral inflammation and associated changes in the brain.

## CLINICAL EVIDENCE: INFLAMMATION AND DEPRESSION

There is a wealth of evidence suggesting that inflammation is associated with depression in the general population, including in adolescents [25]. Longitudinal studies have shown that concentrations of inflammatory markers such as CRP and TNF-α can predict changes in depressive symptoms in adolescents [26,27]. These findings have been corroborated in recent systematic reviews which highlighted bidirectional associations between inflammation and depression in young people [28]. A meta-analysis indicated that depression may significantly predict future inflammation [*r* = 0.29, 95% confidence interval (CI) = 0.04–0.50] and conversely inflammation may significantly predict future depression, though the magnitude of this effect is small (*r* = 0.04, 95% CI = 0.002–0.08) [29]. Using RNA sequencing in whole blood, a recent study in Brazil has also shown that inflammation-related biological pathways such as influenza-linked hypercytokinemia or hyperchemokinemia, interferon signalling, and complement signalling were upregulated in adolescents with MDD compared to those without MDD, but only in adolescent girls [30^▪^]. Although the majority of research in this field focuses on peripheral measures of inflammation, studies involving adolescents have shown that peripheral TNF-α concentration is associated with alterations in resting state functional connectivity in the brain [31], whereas peripheral IL-6 in depressed adolescents is positively associated with glutamate in the anterior cingulate cortex [32]. Thus, peripheral inflammatory biomarkers may still provide some insights into changes in brain development, function, or chemistry in the absence of direct measures of neuroinflammation.

Beyond simply being associated with depression, there is some evidence to suggest that inflammation may contribute mechanistically to the development of depression. Adolescents with first-episode major depressive disorder (MDD) show differences in inflammatory biomarker concentrations compared to controls, and these differences are attenuated after initiation of antidepressant treatment [33]. Crucially, a recent meta-analysis found a small but significant (standardized mean difference = –0.29) effect of anti-inflammatory interventions on reducing depressive symptoms in adolescents, suggesting that inflammation may be a mediating mechanism for depression amongst adolescents [34^▪^]. Potential anti-inflammatory interventions for depression should thus be tested in adolescents with HIV who exhibit chronic low-grade inflammation.

Several studies have also found associations between inflammatory biomarkers and depression in people with HIV, though these have largely involved older adults. Between 2013 and 2022, 33 studies were published which explored this question, and these were assessed in a scoping review [35]. A proportion of these studies reported significant associations for the pro-inflammatory cytokines IL-6 (7 of 17 studies) and TNF-α (5 of 11 studies) with depressive symptoms in people with HIV. More recently, Hussain *et al.*[36] measured peripheral inflammation in older adults with HIV and found that higher D-dimer concentrations (indicative of coagulation imbalance, which is linked to the inflammatory response) were associated with depressive symptoms, but only for participants reporting higher levels of loneliness. Thus, there is increasing evidence to suggest that inflammation may be associated with depression in at least a subset of adults with HIV.

We recently investigated the mediating role of peripheral and CNS inflammation on the relationship between HIV status and depressive symptoms in adolescents living in Cape Town, South Africa [37^▪^]. In a small sample (*n* = 60, median age 15.5 years), we observed significant differences between adolescents with HIV compared to those without (and unexposed to) HIV in markers of neuroinflammation (choline concentration in the basal ganglia, measured using magnetic resonance spectroscopy) and peripheral inflammation (MCP-1, YKL-40 and IL-1β in blood serum). However, these inflammatory biomarkers were not found to be significantly associated with depressive symptom severity, likely due to the small sample size and low prevalence of depression overall, as only 35% of participants met criteria for moderate or severe depressive symptoms [38]. Consequently, we did not detect significant mediation effects of these inflammatory biomarkers on the association between HIV status and depressive symptoms in this sample. Further work in larger cohorts with clinically significant depressive symptom severity is necessary to fully characterize the relationship between inflammation and depression in adolescents with HIV.

## CLINICAL EVIDENCE: INFLAMMATION AND ANXIETY DISORDER

Young people with anxiety disorder may exhibit peripheral inflammation, though there are conflicting findings across studies. A recent meta-analysis, which included nine studies investigating inflammation in children and adolescents, did not find overall significant differences in inflammatory cytokines or CRP between participants with and without anxiety disorders [39]. The authors suggested that this lack of significant differences may be attributed to small sample sizes and high levels of heterogeneity across publications. Moreover, it may be useful to explore other measures of inflammation in this context. For instance, haematological parameters such as neutrophil:lymphocyte ratio and monocyte:lymphocyte ratio were found to be higher in children and adolescents with anxiety disorder than those without [40]. Similarly, serum concentrations of the neurotrophic factor S100B, an astrocytic protein whose peripheral concentrations are considered a marker of blood-brain barrier disruption but which has been less explored in previous studies of mental health risk, were significantly lower in people with anxiety, though this study involved older adults [41]. Thus, while the relationship between inflammation and anxiety disorders in adolescents remains unresolved, future studies may explore novel biomarkers beyond cytokines and chemokines.

Although specific evidence in people with HIV is sparse, a recent study compared inflammatory markers in a small sample of adult men with HIV in Beijing, China, some of whom had anxiety disorder while others did not [42^▪^]. Participants with anxiety in this study showed higher eotaxin (pro-inflammatory chemokine) and higher IL-4 (anti-inflammatory cytokine) concentrations in blood plasma than participants without anxiety. The observed increases in inflammatory proteins with opposing functions indicates that further work is necessary to resolve the relationship between inflammatory biomarkers and anxiety disorders in people with HIV. No studies have yet explored this relationship in adolescents with HIV, representing a major knowledge gap.

## FUTURE DIRECTIONS

Few studies have directly investigated the role of inflammation in mental health issues amongst people with HIV, and those focusing on adolescents with HIV are even more sparse. Nevertheless, robust evidence is emerging in psychiatric research which points to inflammation as a significant contributor to mental health issues in a subset of the general population. Given that people with HIV are consistently found to exhibit low-grade systemic and CNS inflammation, and that adolescents with perinatally-acquired HIV may have experienced this neuroinflammation through critical developmental periods, it is important to determine whether the increased risk for mental health issues in this population may be at least partly driven by HIV-related neuroinflammation.

**FIGURE 1:**
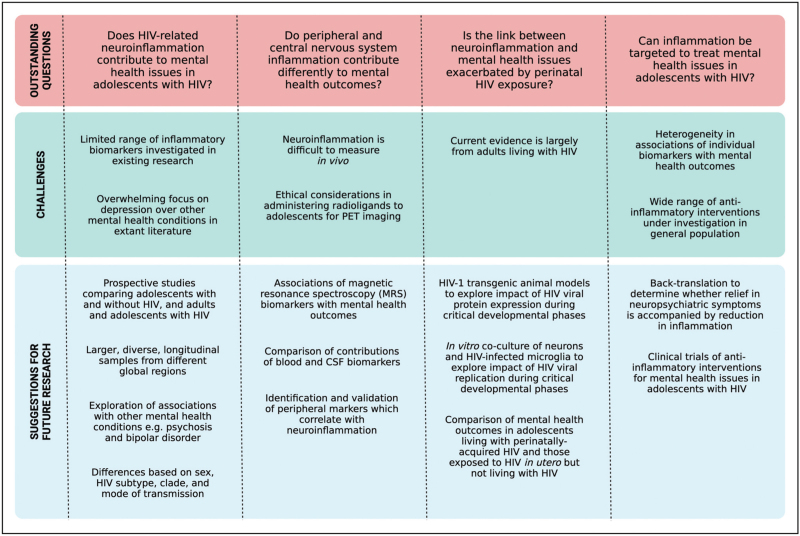
Key outstanding questions concerning the role of inflammation in mental health outcomes amongst adolescents with HIV, and possible approaches to answering these questions. Figure created with BioRender.

Figure 1 highlights key outstanding questions and possible approaches for future studies to answer these questions. Given the paucity of research in this field, the first priority must be to establish whether inflammation contributes to mental health outcomes (including under-explored conditions in this context, such as psychosis and bipolar disorder) in large, diverse, and global cohorts of adolescents with HIV. There is also a need to parse out the effects of systemic and CNS inflammation on mental health outcomes, which may be accomplished by studies using neuroimaging biomarkers of inflammation measured using techniques such as magnetic resonance spectroscopy. With further validation of biomarkers (both peripheral and central) that reliably predict mental health outcomes in young people with HIV, stratified clinical trials may be carried out to test the efficacy of anti-inflammatory interventions to prevent or treat mental health issues in this population.

It will also be useful to investigate whether early-life exposure to HIV and ART (overlapping with key neurodevelopmental periods) results in greater risk for mental health issues, especially when compared to HIV acquired later in life. This relationship may be characterized through studies of mental health outcomes in adolescents with perinatally-acquired HIV compared to those with recently-acquired HIV, and adolescents with perinatally-acquired HIV compared to HEU adolescents. Mental health issues in adolescents with HIV are also associated with poorer adherence and an increased risk of viral nonsuppression [43], hence the neuroinflammatory consequences of ART interruption (particularly early in life) and any subsequent links to mental health outcomes should also be explored.

## CONCLUSION

There is strong evidence to suggest that neuroinflammation and peripheral inflammation may play a role in the development of mental health issues in adolescents, but research in adolescents with HIV is sparse. Research cohorts of children and adolescents with HIV regularly measure inflammatory biomarkers, and are increasingly measuring mental health outcomes as well. It is critical to capitalize on these opportunities to investigate the potential contribution of HIV-related inflammation to a wide range of mental health issues in these cohorts. Fully characterizing the relationship between inflammation and mental health in adolescents with HIV may help improve the prediction, prevention, early intervention, and treatment of mental health issues in this population.

## Acknowledgements

*None.*

### Financial support and sponsorship

*A.M.R.L. was supported by funding from the Wellcome Trust (Grant Number 218493/Z/19/Z).*

### Conflicts of interest

*A.M.R.L., J.H.V. and B.L. have no conflicts of interest to disclose.*

## REFERENCES AND RECOMMENDED READING

Papers of particular interest, published within the annual period of review, have been highlighted as:

▪ of special interest
▪▪ of outstanding interest
